# Exploring Whether the Electronic Optimization of Routine Health Assessments Can Increase Testing for Sexually Transmitted Infections and Provider Acceptability at an Aboriginal Community Controlled Health Service: Mixed Methods Evaluation

**DOI:** 10.2196/51387

**Published:** 2023-11-30

**Authors:** Heather McCormack, Handan Wand, Christy E Newman, Christopher Bourne, Catherine Kennedy, Rebecca Guy

**Affiliations:** 1 Kirby Institute University of New South Wales Kensington Australia; 2 Centre for Population Health New South Wales Ministry of Health Sydney Australia; 3 Centre for Social Research in Health University of New South Wales Kensington Australia; 4 Sydney Sexual Health Centre Sydney Australia; 5 Maari Ma Health Aboriginal Corporation Broken Hill Australia

**Keywords:** sexual health, sexually transmitted infection, STI, primary care, Indigenous health, electronic medical record, EMR, medical records, electronic health record, EHR, health record, health records, Indigenous, Native, Aboriginal, sexual transmission, sexually transmitted, time series, testing, uptake, acceptance, acceptability, adoption, syphilis, sexually transmitted disease, STD, systems change, health assessment, health assessments, prompt, prompts, implementation, youth, young people, adolescent, adolescents

## Abstract

**Background:**

In the context of a syphilis outbreak in neighboring states, a multifaceted systems change to increase testing for sexually transmitted infections (STIs) among young Aboriginal people aged 15 to 29 years was implemented at an Aboriginal Community Controlled Health Service (ACCHS) in New South Wales, Australia. The components included electronic medical record prompts and automated pathology test sets to increase STI testing in annual routine health assessments, the credentialing of nurses and Aboriginal health practitioners to conduct STI tests independently, pathology request forms presigned by a physician, and improved data reporting.

**Objective:**

We aimed to determine whether the systems change increased the integration of STI testing into routine health assessments by clinicians between April 2019 and March 2020, the inclusion of syphilis tests in STI testing, and STI testing uptake overall. We also explored the understandings of factors contributing to the acceptability and normalization of the systems change among staff.

**Methods:**

We used a mixed methods design to evaluate the effectiveness and acceptability of the systems change implemented in 2019. We calculated the annual proportion of health assessments that included tests for chlamydia, gonorrhea, and syphilis, as well as an internal control (blood glucose level). We conducted an interrupted time series analysis of quarterly proportions 24 months before and 12 months after the systems change and in-depth semistructured interviews with ACCHS staff using normalization process theory.

**Results:**

Among 2461 patients, the annual proportion of health assessments that included any STI test increased from 16% (38/237) in the first year of the study period to 42.9% (94/219) after the implementation of the systems change. There was an immediate and large increase when the systems change occurred (coefficient=0.22; *P*=.003) with no decline for 12 months thereafter. The increase was greater for male individuals, with no change for the internal control. Qualitative data indicated that nurse- and Aboriginal health practitioner–led testing and presigned pathology forms proved more difficult to normalize than electronic prompts and shortcuts. The interviews identified that staff understood the modifications to have encouraged cultural change around the role of sexual health care in routine practice.

**Conclusions:**

This study provides evidence for the first time that optimizing health assessments electronically is an effective and acceptable strategy to increase and sustain clinician integration and the completeness of STI testing among young Aboriginal people attending an ACCHS. Future strategies should focus on increasing the uptake of health assessments and promote whole-of-service engagement and accountability.

## Introduction

### Background

Aboriginal and Torres Strait Islander people in Australia can access sexually transmitted infection (STI) testing at primary health care services, including Aboriginal Community Controlled Health Services (ACCHSs) and mainstream general practice or government sexual health services [1]. The first ACCHS was established in 1971; now, there are >150 ACCHSs nationwide [2]. As in other countries with a history of colonization similar to that of Australia, culturally competent primary health care services run by and for Aboriginal communities play a vital role in ensuring equitable and culturally safe access to sexual and reproductive health care [3-5]. Reflecting this, previous research has identified that ACCHSs are more likely to provide health care that is free of racism than mainstream services [6]. ACCHSs are well situated to embed screening for STIs into routine clinical practice to more regularly identify asymptomatic infections [7].

In Australia, the highest rates of notified STIs (chlamydia, gonorrhea, and syphilis) are among young people aged 15 to 29 years, higher in young Aboriginal and Torres Strait Islander people than in young non-Indigenous people, and the highest in remote areas [8]. *Chlamydia trachomatis* (CT) is the most commonly reported STI among young Aboriginal people, followed by *Neisseria gonorrhea* (NG) and *Treponema pallidum* (syphilis) [9]. CT and NG are easily cured with antibiotics but can be asymptomatic and lead to complications if not treated, such as pelvic inflammatory disease, ectopic pregnancy, and infertility [10-13]. Syphilis is of major concern in Australia, with an expanding epidemic over the last decade beginning in northern Australia and extending to southern Australia, including New South Wales (NSW) and Victoria. Nationally, the notification rate (defined as the rate of notifiable diagnoses per 100,000 person years) is 6 times higher for Aboriginal and Torres Strait Islander people and up to 50 times higher in very remote areas of northern Australia [8]. If left untreated, syphilis can have particularly serious consequences, including miscarriage, stillbirth, and fetal abnormalities [11]. Modeling suggests that the STI prevalence in remote Aboriginal and Torres Strait Islander communities could be considerably reduced if screening was increased to 60% coverage and sustained for 10 years [14], and increasing STI screening rates for young Aboriginal people is a priority target in state and national public health strategies [15,16].

Most ACCHS patients present for reasons other than seeking an STI test; therefore, the mechanisms proposed to increase STI testing in this cohort include integration into other routine primary care visits, such as the Medicare Benefits Schedule item 715 annual health assessment (hereinafter referred to as *health assessment*) [17]. This is a structured preventive health assessment that can be provided annually to Aboriginal and Torres Strait Islander people in primary care settings, including ACCHSs. Australia’s universal health insurance system, Medicare, provides a rebate per individual health assessment, and additional practice-level incentives are attached for eligible practices managing patients with chronic disease [18]. The *National guide to a preventive health assessment for Aboriginal and Torres Strait Islander people* [19] recommends the inclusion of STI screening for sexually active young people, along with an assessment of lifestyle factors; social and emotional well-being; vaccination status; and blood lipids, renal function, and blood glucose levels. Approximately one-quarter of young Aboriginal and Torres Strait Islander people participate in a health assessment each year [20]. This is a national figure, and local uptake is highly variable among regions and services [17]. The uptake of health assessments more than tripled in the decade preceding the COVID-19 pandemic, which resulted in a slight decrease of 2% over 2 years [20]. The pandemic also resulted in many health services temporarily deprioritizing the routine STI screening of asymptomatic patients in a context of significant disruption to clinical services [21].

Health assessments involve completing an electronic template in the patient electronic medical record (EMR), which can be customized at the practice level or by the software vendor [22]. The potential of EMR customization to improve the completeness and consistency of health assessments has been anticipated since ACCHSs first began to implement computerized health assessment templates >10 years ago [23]. Electronic prompts have been found to be more effective at increasing STI test requests made by clinicians in other settings than nonelectronic reminders, such as reminder stickers on paper medical records or pathology result forms [24]. The implementation of electronic prompts and automated pathology sets in both the mainstream general practice setting and at government-funded sexual health clinics has been found to increase comprehensive STI testing in line with clinical guidelines and the resulting detection of STIs for patients considered to be at higher risk, such as gay and bisexual men [25-27]. To our knowledge, the use of electronic prompts or automated pathology sets to increase STI testing in the context of a health assessment at ACCHSs has not been evaluated.

The implementation and integration of complex health care interventions into routine clinical practice relies on action and interaction by and among individuals—the “work” of normalizing and embedding change [28]. In this paper, normalization is defined as per May et al [29] as the work of actors when engaging with new or changed activities by which the new activities become routinely embedded into existing knowledge and practices. Through this lens, implementation is characterized as an inherently social process involving collective action from human actors within dynamic contexts [30]. This provides a framework to analyze the factors that encourage or inhibit the incorporation of health care interventions into routine clinical practice [31].

### Objectives

This mixed methods evaluation examined whether the systems change implemented at an NSW ACCHS increased the integration of STI testing into health assessments in young people, with a particular focus on concurrent syphilis testing, and explored the understandings of the acceptability and normalization of the systems change among ACCHS staff.

## Methods

### Setting

This study was conducted in partnership with a western NSW ACCHS caring for a large Aboriginal population [32] of between 8.4% in the main town center and 61.2% in smaller satellite towns (compared with 2.9% for NSW as a whole) [33,34]. This ACCHS provides 53,000 occasions of service to an average of 3500 patients per year, 80% of whom are Aboriginal patients. This figure accounts for all separate interactions with patients and includes face-to-face visits and telephone calls, both clinical and administrative. As the Torres Strait Islander population is low, we will hereinafter respectfully use the term *Aboriginal* when referring to this setting. Although NSW is home to the largest Aboriginal population in Australia, there is little quantitative sexual health research conducted with Aboriginal populations in NSW [35], although some qualitative studies have been published in recent years [36,37].

### The Systems Change

We collaboratively developed a multifaceted systems change with ACCHS staff in March 2019. ACCHS clinical, managerial, and administrative staff who had been identified as potential sexual health champions cocreated the systems change at a workshop facilitated by 2 members of the research team (HM and CB). The workshop aimed to identify strategies to improve syphilis testing rates and STI testing rates more broadly among the patient population within the existing range of services, activities, and staff skills available. A core component was the addition to the EMR of an electronic prompt and an automated pathology set. The prompt was attached to the health assessment template completed by general practitioners (GPs) and triggered for all patients in the target age range, advising the GP to query sexual activity since the patient’s last test because guidelines recommend STI screening after a change in partner or otherwise annually [38]. An affirmative indication updated the health assessment template in the EMR and autopopulated a pathology request for a comprehensive STI test (chlamydia, gonorrhea, and syphilis). The automated pathology set replaced the manual completion of pathology requests for individual STI tests with a single-click button that autopopulated a pathology request for a comprehensive STI test. The prompt aimed to increase the integration of STI tests into the health assessment, and the automated pathology test set aimed to increase the inclusion of syphilis tests when STI tests were requested, both within and outside of the health assessment.

Three other operational components completed the package: credentialing registered nurses (RNs) and Aboriginal health practitioners (AHPs) to undertake STI screening, pathology request forms presigned by a GP to authorize tests conducted by RNs and AHPs [39], and the enhanced internal reporting of STI screening targets to staff via standing agenda items at staff meetings for detailed data reports. Credentialing RNs and AHPs aimed to create opportunities for STI testing in clinical encounters that did not involve a GP, whereas having pathology forms presigned by a GP allowed these tests to receive Medicare funding under the signing GP’s provider number. Enhanced data reporting has been shown to be an important component of continuous quality improvement (CQI) systems implemented at ACCHSs [40,41]. These 3 components aimed to increase STI testing outside of the health assessment.

### Strengths-Based Approach

Our evaluation explicitly adopted a strengths-based approach. This project centers the strengths, assets, and capabilities of ACCHSs and ACCHS staff [42], rather than deficit-focused epidemiological framing that can act as a barrier to improving health outcomes [43]. As such, both the qualitative and quantitative analyses focused on factors associated with positive health outcomes for Aboriginal people rather than measuring ill health against a non-Indigenous baseline [44], and the integration of findings in the discussion foregrounded the strengths and enablers of success rather than barriers and deficiencies.

### Mixed Methods Approach

Our mixed methods evaluation used an explanatory sequential design as defined by Creswell and Clark [45], with quantitative data collected first and then used to inform qualitative data collection and analysis. This design supported the use of in-depth qualitative analysis to more closely examine potential explanations for the quantitative findings, which provides greater insight into the performance of the systems change than quantitative analysis alone [45]. The interview guide for the qualitative component was informed by preliminary findings from the quantitative component, with the qualitative interviews then exploring how staff understood the implementation and outcomes of the systems change. Our qualitative analysis allowed the researchers to explore how and why the components of the systems change had or had not been successfully normalized into staff experiences of routine practice, building on our quantitative analysis to provide a more whole and complex picture of the success of the systems change. The quantitative and qualitative findings have been narratively integrated and explored together in the *Discussion* section.

### Quantitative Methods

Our analysis was based on an annually deduplicated data set of unique Aboriginal patients aged 15 to 29 years who attended the NSW ACCHS in the 24 months before implementation and in the 12 months after implementation (April 2017-March 2020). Using the exact probabilities of the binominal distribution, we determined that a sample size of ≥2000 patients would be sufficient to detect a difference of 5% to 7% in the proportion of those who had an STI test between the intervention and control periods, assuming that STI testing coverage was 10% to 20%, with 80% power.

The primary outcome for the quantitative component of this study was the proportion of health assessments that included a test for any STI (chlamydia, gonorrhea, or syphilis). The secondary outcomes were the proportion of health assessments that included all 3 STI tests, the proportion of health assessments inclusive of a chlamydia and gonorrhea test that also included a syphilis test (concurrent testing), the proportion of the total study population that received a test for any STI (chlamydia, gonorrhea, or syphilis), and the proportion of the total study population that received a health assessment. The control outcome was the proportion of health assessments that included a random blood glucose level test, a recommended inclusion in the health assessment unaffected by the systems change.

**Figure 1:**
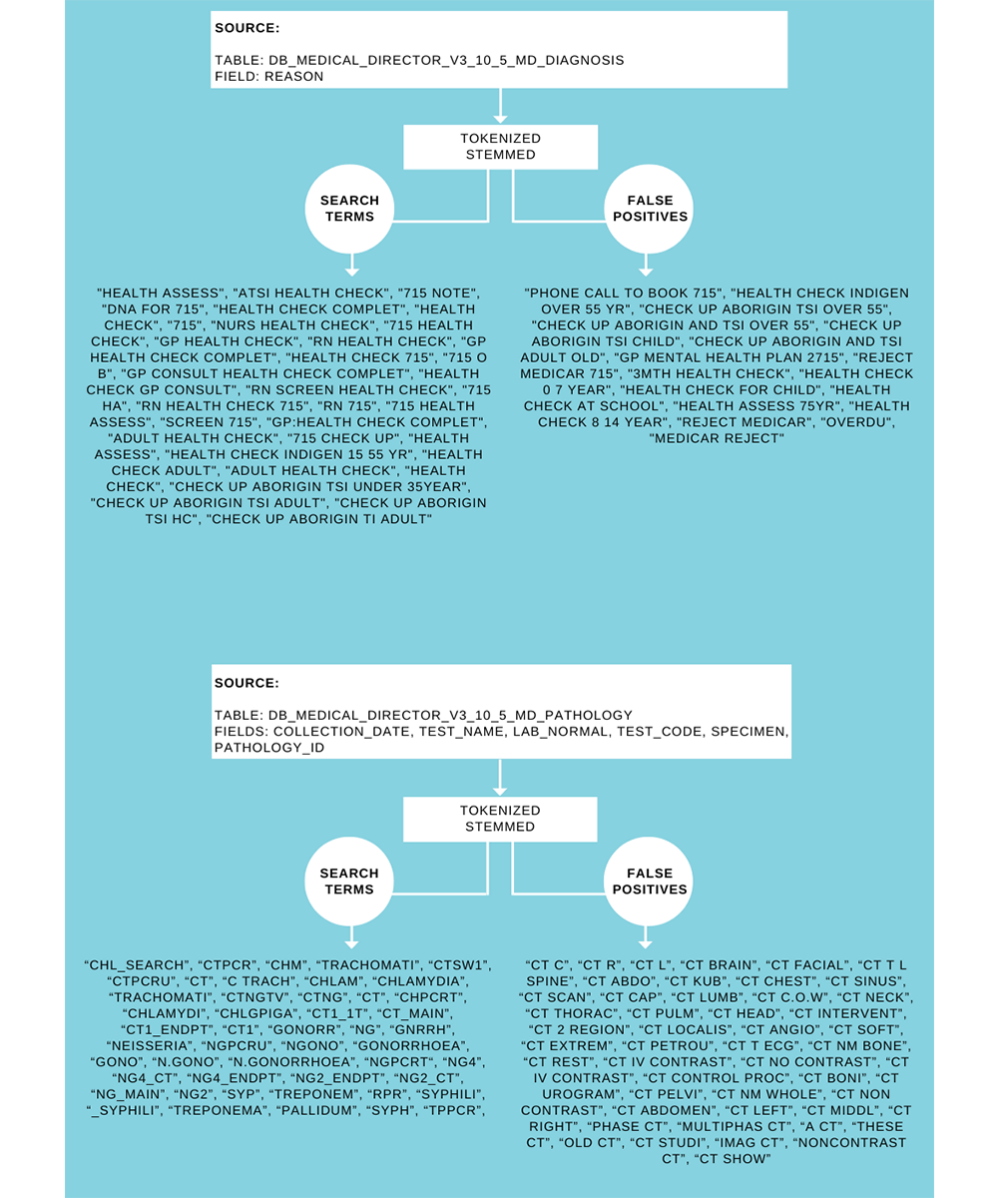
Health assessment and sexually transmitted infection test request data extract coding.

Under strict confidentiality and privacy protocols, deidentified routinely collected clinical data for all young Aboriginal people aged 15 to 29 years who attended the ACCHS in the study period (April 2017-March 2020) were extracted from the EMR using software called GRHANITE (Generic Health Network Information Technology for the Enterprise) [46] via the enrolment of the service in the ATLAS sentinel surveillance network, which has been described elsewhere [47]. We estimated the average number of clinical interactions per person and their 95% CIs using Poisson regression. Demographic variables included age, sex, and Aboriginal status. Testing variables collected via ATLAS included the test performed (chlamydia, gonorrhea, or syphilis) and the date the test was conducted. A further testing variable (random blood glucose level test) was collected manually by the in-house ACCHS statistician because it was outside the scope of ATLAS. ATLAS data collected via natural language processing and keyword search was validated with the help of ACCHS clinical staff. The ATLAS keywords are shown in Figure 1. As tests were conducted at the time of consultation at an in-house pathology service, the date of the test request is not reported separately. Attendance and visit variables included the date of consultation and Medicare Benefits Schedule item for health assessment.

Annual changes in each outcome were examined by age group and sex. An interrupted time series (ITS) regression model (also known as segmented regression) was used to estimate the changes in the proportion of any STI test conducted among those who had a health assessment over time. Regression coefficients with 95% CIs were presented for comparing the before and after periods. An ITS is a technique used for evaluating what are sometimes referred to as natural experiments by estimating the consequence of specific changes in a real-world setting and adjusting for individual- and community-level factors via multilevel regression models to account for covariates [48]. The ITS analysis was then stratified by sex for the primary outcome and the control outcome.

### Qualitative Methods

The staff of the NSW ACCHS were the population for the qualitative component of this study. Stratified purposeful sampling [49] was used to recruit staff, who were informed of the project at an in-service information program delivered by members of the research team (HM and CB) and then invited to participate via email, with key staff members also contacted by telephone. Recruitment remained open until the sample contained representation from each of the 3 inclusion categories of clinical staff (GPs, RNs, and AHPs). Because of the impacts of the COVID-19 pandemic, interviews were not completed until the year after the implementation of the systems change. Each participant completed a semistructured interview lasting approximately 1 hour that followed an interview guide informed by normalization process theory (NPT) [28,30,50]. Previous sexual health research has drawn on NPT to examine provider-initiated HIV testing and counseling in South Africa [51] and annual STI screening in remote ACCHSs in the Northern Territory, Australia [52,53]. As well as open-ended questions on topics such as how the systems change affected work practices, the factors that made the components of the systems change easier or more difficult to implement, perceived impacts on the service, and suggestions for future modifications, the interviews included questions informed by the quantitative findings. Participants were asked whether they thought that STI testing rates had increased after the systems change, then presented with preliminary quantitative data and asked for their opinions on how the components of the systems change had contributed to these findings.

The interviews were transcribed and analyzed thematically by the first author (HM) as per Braun and Clarke [54] and later works [55,56] using NVivo software (Lumivero). HM is an Aboriginal woman from NSW with expertise in sexual health. She was supported throughout the analysis by CN, a non-Indigenous researcher with substantial experience in qualitative approaches to sexual health research. Codes were informed by NPT [31,50], focusing on the 4 main constructs of coherence, cognitive participation, collective action, and reflexive monitoring. Component definitions within each construct were informed by the study conducted by Hengel et al [53], which used NPT to assess the integration into routine practice of an ACCHS CQI program. Textbox 1 (adapted from Hengel et al [53] and Murray et al [31]) outlines NPT constructs and components.

**Normalization process theory constructs and components.**

**Construct, component, and definition of component**

- Coherence
  Differentiation: perceived difference from existing practice
  Communal specification: shared understanding of the aims of the systems change
  Individual specification: individual understanding of the aims of the systems change
  Internalization: perceived importance and value of the systems change
- Cognitive participation
  Initiation: willingness to drive the systems change forward
  Enrolment: collaborative work to make the systems change succeed
  Legitimation: perceptions of the worthiness of time and effort
  Activation: likelihood of participants sustaining the changes to work practice
- Collective action
  Interactional workability: effect of the systems change on shared work
  Relational integration: accountability built on knowledge
  Skill set workability: appropriateness of the systems change to participant skill sets
  Contextual integration: compatibility with existing policies, practices, and environmental context
- Reflexive monitoring
  Systematization: effectiveness of the systems change in daily practice
  Communal appraisal: group evaluation of the systems change
  Individual appraisal: personal relationship with the systems change
  Reconfiguration: opportunities for participant experience to inform modifications to systems change

HM developed a thematic map and undertook a reread of the raw data to recode any data that were missed in the earlier stages of analysis. Codes, themes, and the analytic framework were discussed by all authors throughout the coding and analysis process to make sense of shared understandings and to interrogate assumptions and interpretations.

### Ethical Considerations

The evaluation was overseen by a research governance committee with representation from the medical staff, nursing staff, public health staff, and primary health care management staff of the ACCHS. An ACCHS staff member also contributed to the research team. This study received ethics approval through the ethics committee of the Aboriginal Health & Medical Research Council of NSW (HREC 1700/20). Participants were informed both at the in-service program and before each interview that their participation was voluntary and provided with a participation information sheet and consent form, the contents of which were read aloud at the commencement of the interview. Informed voluntary consent was obtained before the collection of any personal information. The research team includes 2 researchers with an existing relationship with the health service with no authoritative influence over prospective interview participants.

## Results

### Quantitative Results

**Figure 2:**
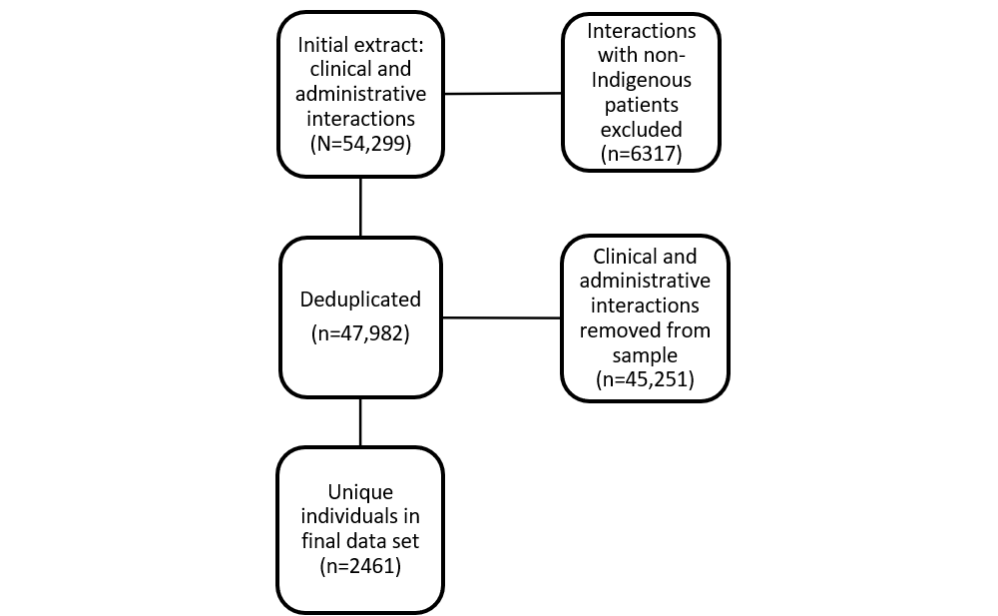
Study population.

During the study period (April 2017-March 2020), a total of 54,299 patient interactions were recorded in the EMR; after excluding interactions recorded with non-Indigenous patients (n=6317, 11.63%) and deduplication, 2461 (4.53%) unique Aboriginal patients who attended the ACCHS for a clinical interaction at least once, with an average of 19 interactions (SE 0.09; 95% CI 19.32-19.68) recorded in the EMR per patient, were included in the analysis. The recorded interactions per patient may have occurred at a single visit or across multiple visits. Among these 2461 unique Aboriginal patients, 644 (26.17%) had at least 1 episode of a health assessment annually recorded as a clinical interaction in the EMR. Of the 2461 unique Aboriginal patients, 45.43% (n=1118) were men, and 54.57% (n=1343) were women, with a median age of 22 (IQR 18-26) years. The study population is shown in Figure 2.

Overall, 27.2% (175/644) of the health assessments included any STI test, 23.6% (152/644) included a CT/NG test, and 18.5% (119/644) included a syphilis test. Combined CT/NG and syphilis testing occurred in 14.9% (96/644) of the health assessments.

Compared with the 24 months before implementation, the annual proportion of health assessments that integrated any STI test (CT/NG and syphilis) increased in the postintervention period (year 1: 38/237, 16%; year 2: 43/188, 22.9%; and intervention period: 94/219, 42.9%). Greater increases were observed in those aged 20 to 24 years and 25 to 29 years than in those aged 15 to 19 years. The increase by age group was similar for all study outcomes. The changes in the integration of a CT/NG test into a health assessment were similar for both male individuals and female individuals, although male individuals had a higher baseline value. However, the increase in concurrent syphilis testing with CT/NG testing was more substantial for male individuals than for female individuals. Similar results by patient sex were observed for the integration of any STI test and the integration of both a CT/NG test and a syphilis test (Table 1).

Of the health assessments that included a CT/NG test, 63.2% (96/152) overall also included a concurrent syphilis test.

**Table 1:** Proportion of health assessments conducted on young Aboriginal people aged 15 to 29 years during the study period that included tests for *Chlamydia trachomatis* (CT) and *Neisseria gonorrhea* (NG) or syphilis (n=644).

Time period and breakdown			Any STI^a^ test included (CT or NG or syphilis; n=175), n (%)	CT and NG test included (n=152), n (%)	Syphilis test included (n=119), n (%)	Combined CT and NG and syphilis testing included (n=96), n (%)
**April 2017-March 2018 (year 1; n=237)**						
	**Breakdown by age group (years)**					
		15-19 (n=86)	14 (16.3)	12 (14)	6 (7)	4 (4.7)
		20-24 (n=81)	13 (16.1)	10 (12.4)	6 (7.4)	3 (3.7)
		25-29 (n=70)	11 (15.7)	10 (14.3)	7 (10)	6 (8.6)
	**Breakdown by sex**					
		Male (n=119)	23 (19.3)	21 (17.7)	9 (7.6)	7 (5.9)
		Female (n=118)	15 (12.7)	11 (9.3)	10 (8.5)	6 (5.1)
**April 2018-March 2019 (year 2; n=188)**						
	**Breakdown by age group (years)**					
		15-19 (n=67)	13 (19.4)	12 (17.9)	5 (7.5)	4 (6)
		20-24 (n=63)	17 (27)	16 (25.4)	12 (19.1)	11 (17.5)
		25-29 (n=58)	13 (22.4)	11 (19)	9 (15.5)	7 (12.1)
	**Breakdown by sex**					
		Male (n=89)	22 (24.7)	21 (23.6)	14 (15.7)	13 (14.6)
		Female (n=99)	21 (21.2)	18 (18.2)	12 (12.1)	9 (9.1)
**April 2019-March 2020 (intervention; n=219)**						
	**Breakdown by age group (years)**					
		15-19 (n=79)	24 (30.4)	20 (25.3)	18 (22.8)	14 (17.7)
		20-24 (n=69)	34 (49.3)	32 (46.4)	25 (36.2)	23 (33.3)
		25-29 (n=71)	36 (50.7)	29 (40.9)	31 (43.7)	24 (33.8)
	**Breakdown by sex**					
		Male (n=113)	55 (48.7)	49 (43.4)	49 (43.4)	43 (38.1)
		Female (n=106)	39 (36.8)	32 (30.2)	25 (23.6)	18 (17)

^a^STI: sexually transmitted infection.

The annual proportion of health assessments inclusive of a CT/NG test that also included a syphilis test increased in all age groups, although those aged 25 to 29 years had higher baseline values than those aged 15 to 19 years or 20 to 24 years (Table 2). The inclusion of a syphilis test in health assessments with a CT/NG test increased for male individuals to 88% (18/32) but remained relatively stable for female individuals.

The annual proportion of young people who received a health assessment fluctuated slightly, with uptake slightly higher in male individuals than in female individuals and in those aged 15 to 19 years than in older young people throughout the study period. Among unique young Aboriginal people attending the service each year, there was a small increase in the proportion tested for CT/NG (from 195/800, 24.4% to 243/823, 29.5%) and a greater increase in the proportion tested for syphilis (from 103/800, 12.9% to 225/823, 27.3%), with increases greater for male individuals than for female individuals for both as well as the outcome: receiving all STI tests (Table 3).

**Figure 3:**
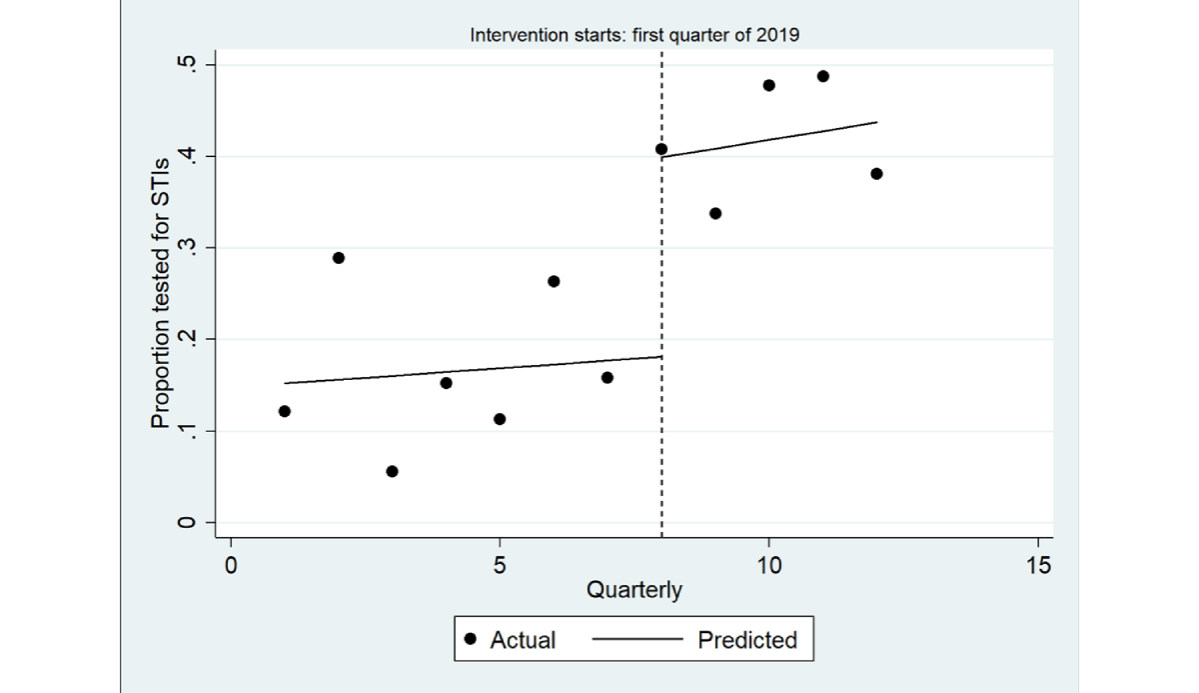
Interrupted time series analysis of health assessments inclusive of any sexually transmitted infection (STI) test (chlamydia, gonorrhea or syphilis): overall study population. Regression with Newey-West SEs: lag(1).

**Figure 4:**
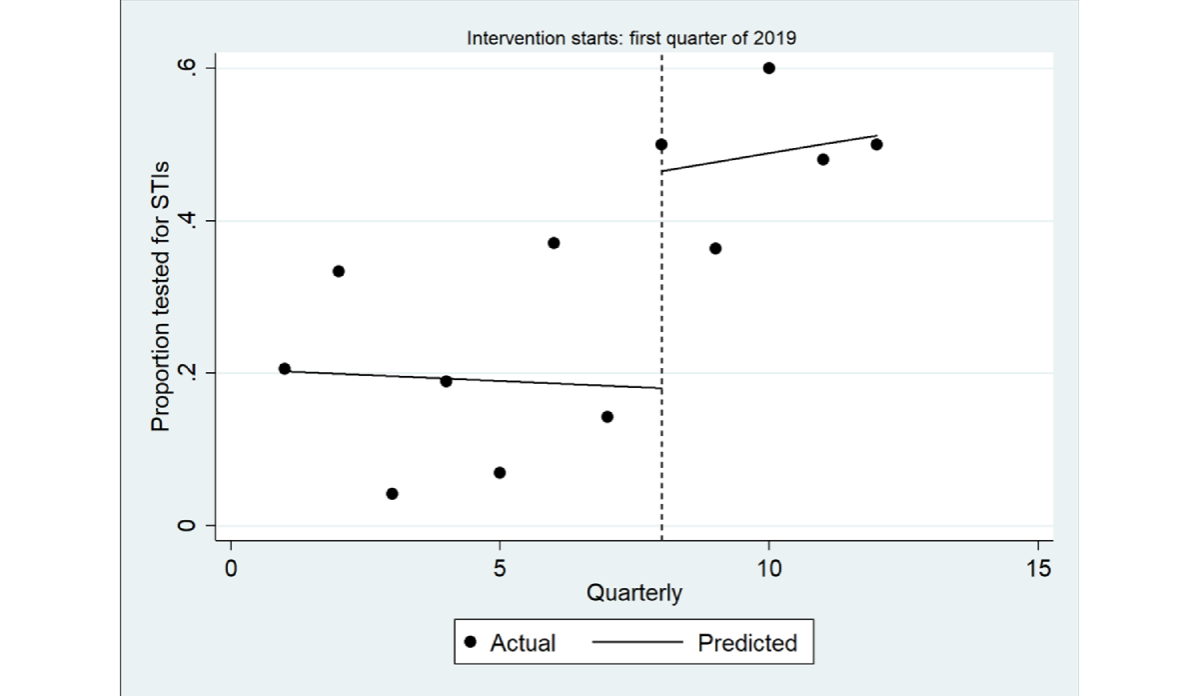
Interrupted time series analysis of health assessments inclusive of any sexually transmitted infection (STI) test (chlamydia, gonorrhea or syphilis): male. Regression with Newey-West SEs: lag(1).

**Figure 5:**
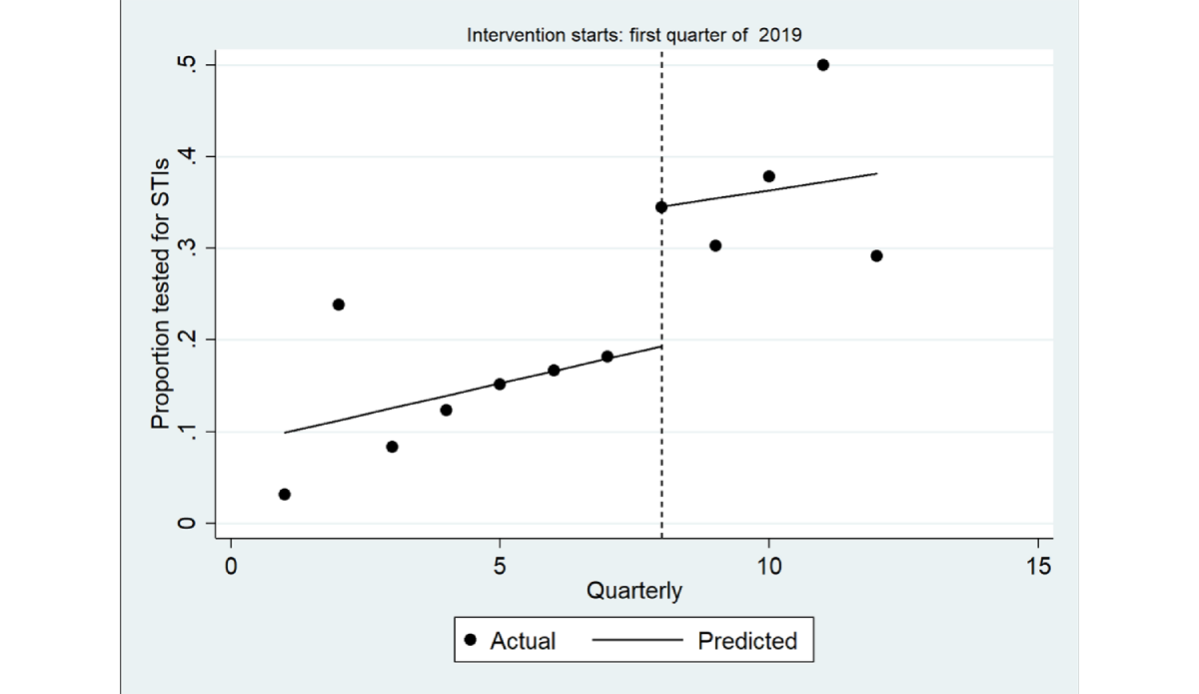
Interrupted time series analysis of health assessments inclusive of any sexually transmitted infection (STI) test (chlamydia, gonorrhea or syphilis): female. Regression with Newey-West SEs: lag(1).

The ITS model shown in Figure 3 shows a significant increase in the quarterly proportion of health assessments completed with young Aboriginal people that included an STI test immediately after implementation (coefficient=0.22; *P*=.003), greater among male individuals (coefficient=0.28; *P*=.01) than among female individuals (coefficient=0.15; *P*=.005; Table 4; Figures 4 and 5). There was no trend in the quarterly intervals in the postintervention period for either male or female individuals.

When the ITS analysis was repeated with the final quarter excluded (January-March 2020, the beginning of the COVID-19 pandemic), an increasing trend was observed (*P*=.006, data not shown).

**Figure 6:**
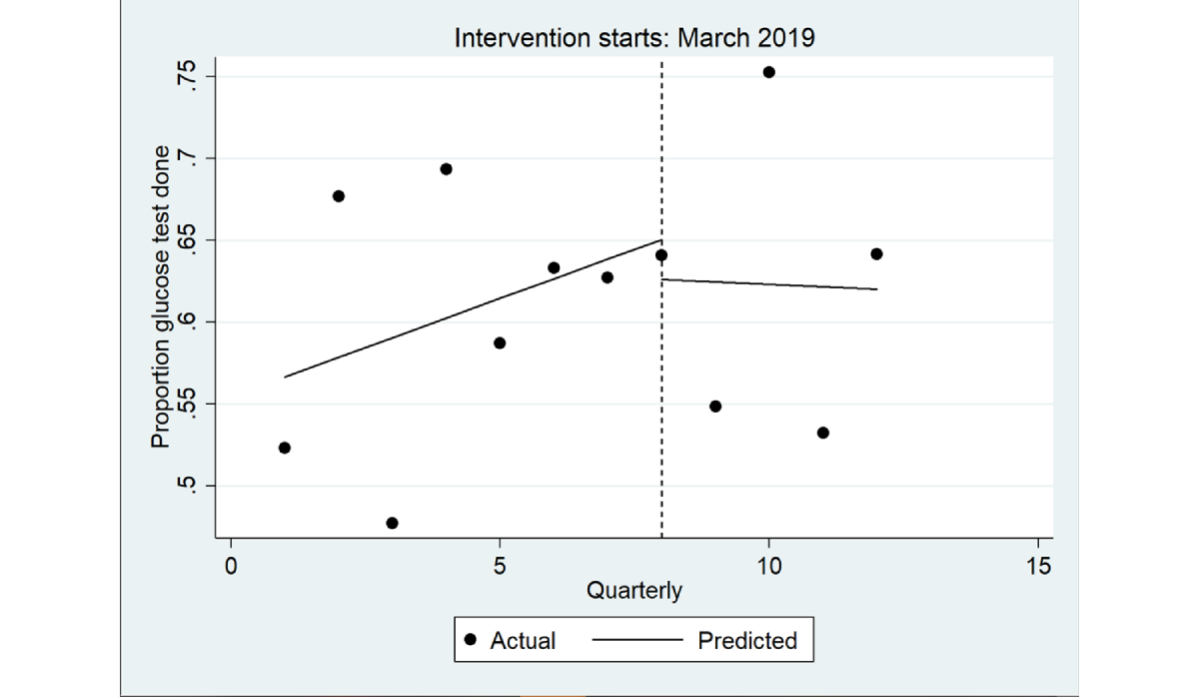
Interrupted time series analysis of health assessments inclusive of a random blood glucose level test: overall study population. Regression with Newey-West SEs: lag(1).

**Figure 7:**
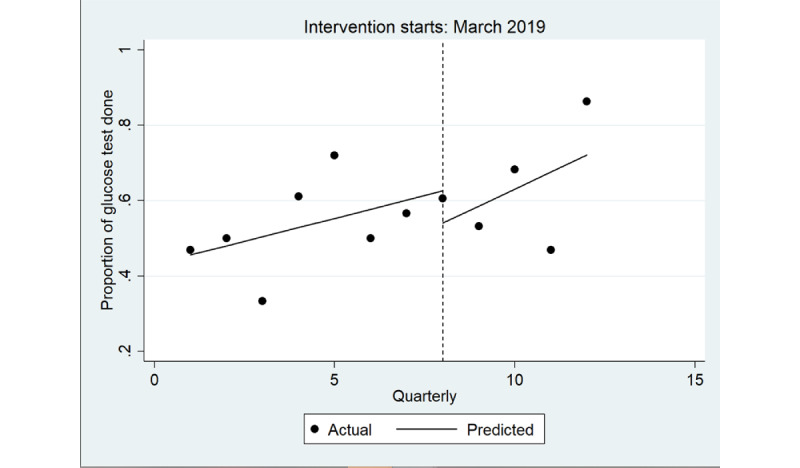
Interrupted time series analysis of health assessments inclusive of a random blood glucose level test: male. Regression with Newey-West SEs: lag(1).

**Figure 8:**
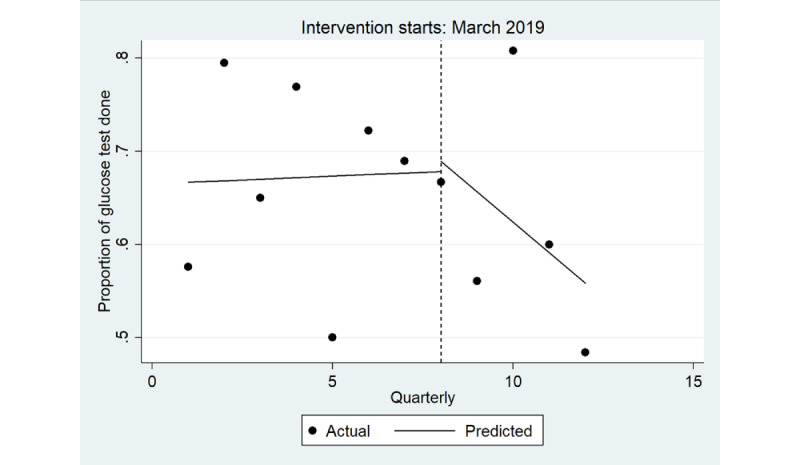
Interrupted time series analysis of health assessments inclusive of a random blood glucose level test: female. Regression with Newey-West SEs: lag(1).

There was no immediate change in the quarterly proportion of health assessments that included a random blood glucose level test (Table 5), overall (Figure 6) and for male individuals (Figure 7) and female individuals (Figure 8). In the postintervention period, no trend was detected overall (*P*=.90) or in male individuals (*P*=.20) and female individuals (*P*=.20).

**Table 2:** Proportion of health assessments conducted in young Aboriginal people aged 15 to 29 years inclusive of a *Chlamydia trachomatis* and *Neisseria gonorrhea* (CT/NG) test that concurrently included a syphilis test (n=152).

Time period and breakdown				Health assessments with a CT and NG test that concurrently also included a syphilis test, n (%)
**April 2017-March 2018 (year 1; n=32)**				
	**Breakdown by age group (years)**			
		15-19 (n=12)	4 (33)	
		20-24 (n=10)	3 (30)	
		25-29 (n=10)	6 (60)	
	**Breakdown by sex**			
		Male (n=21)	7 (33)	
		Female (n=11)	6 (55)	
**April 2018-March 2019 (year 2; n=39)**				
	**Breakdown by age** **group** **(years)**			
		15-19 (n=12)	4 (33)	
		20-24 (n=16)	11 (69)	
		25-29 (n=11)	7 (64)	
	**Breakdown by sex**			
		Male (n=21)	13 (62)	
		Female (n=18)	9 (50)	
**April 2019-March 2020 (intervention; n=81)**				
	**Breakdown by age group (years)**			
		15-19 (n=20)	14 (70)	
		20-24 (n= 32)	23 (72)	
		25-29 (n=29)	24 (83)	
	**Breakdown by sex**			
		Male (n=49)	43 (88)	
		Female (n=32)	18 (56)	

**Table 3:** Proportion of young Aboriginal people aged 15 to 29 years attending the service during the study period who received a sexually transmitted infection (STI) test (chlamydia, gonorrhea, or syphilis) or a health assessment (n=2461).

Time period and breakdown				Received a CT^a^ and NG^b^ test (n=637), n (%)		Received a syphilis test (n=470), n (%)		Received all STI tests (n=330), n (%)		Received a health assessment (n=644), n (%)
**April 2017-March 2018 (year 1; n=800)**										
	**Breakdown by age group (years)**									
		15-19 (n=276)	48 (17.4)		21 (7.6)		14 (5.1)		86 (31.2)	
		20-24 (n=273)	74 (27.1)		40 (14.7)		30 (11)		81 (29.7)	
		25-29 (n=251)	73 (29.1)		42 (16.7)		35 (13.9)		70 (27.9)	
	**Breakdown by sex**									
		Male (n=364)	65 (17.9)		38 (10.4)		32 (8.8)		119 (32.7)	
		Female (n=436)	130 (29.8)		65 (14.9)		47 (10.8)		118 (27.1)	
**April 2018-March 2019 (year 2; n=838)**										
	**Breakdown by age group (years)**									
		15-19 (n=285)	515 (17.9)		295 (10.2)		18 (6.3)		67 (23.5)	
		20-24 (n=280)	79 (28.2)		60 (21.4)		42 (15)		63 (22.5)	
		25-29 (n=273)	69 (25.3)		53 (19.4)		32 (11.7)		58 (21.3)	
	**Breakdown by sex**									
		Male (n=384)	70 (18.2)		43 (11.2)		33 (8.6)		89 (23.2)	
		Female (n=454)	129 (28.4)		99 (21.8)		59 (13)		99 (21.8)	
**April 2019-March 2020 (intervention; n=823)**										
	**Breakdown by age group (years)**									
		15-19 (n=279)	59 (21.2)		50 (17.9)		32 (11.5)		79 (28.3)	
		20-24 (n=280)	95 (33.9)		83 (29.6)		61 (21.8)		69 (24.6)	
		25-29 (n=264)	89 (33.7)		92 (34.9)		66 (25)		71 (26.9)	
	**Breakdown by sex**									
		Male (n=370)	98 (26.5)		94 (25.4)		77 (20.8)		113 (30.5)	
		Female (n=453)	145 (32)		131 (28.9)		82 (18.1)		106 (23.4)	

^a^CT: *Chlamydia trachomatis.*

^b^NG: *Neisseria gonorrhea*.

**Table 4:** Estimates from the interrupted time series analysis for the proportion of health assessments inclusive of any sexually transmitted infection test (chlamydia, gonorrhea, or syphilis).

	Overall			Male			Female	
	Coefficient (95% CI)	*P* value	Coefficient (95% CI)		*P* value	Coefficient (95% CI)		*P* value
Before the intervention period of March 2019	0.004 (−0.02 to 0.03)	.70	−0.003 (−0.04 to 0.03)		.08	0.01 (−0.008 to 0.030)		.18
Immediate effect of the intervention period of March 2019	0.22 (0.10 to 0.34)	.003	0.28 (0.08 to 0.50)		.01	0.15 (0.06 to 0.24)		.005
Overall effect of the intervention period of March 2019	0.01 (−0.02 to 0.04)	.40	0.01 (−0.02 to 0.04)		.40	0.01 (−0.04 to 0.06)		.67

**Table 5:** Estimates from the interrupted time series for the proportion of health assessments that included a random blood glucose level test.

	Overall		Male			Female	
	Coefficient (95% CI)	*P* value	Coefficient (95% CI)	*P* value	Coefficient (95% CI)		*P* value
Before the intervention period of March 2019	0.01 (−0.003 to 0.030)	.10	0.02 (−0.004 to 0.050)	.09	0.002 (−0.03 to 0.03)		.90
Immediate effect of the intervention period of March 2019	−0.02 (−0.11 to 0.07)	.55	−0.09 (−0.31 to 0.14)	.40	0.01 (−0.18 to 0.20)		.90
Overall effect of the intervention period of March 2019	−0.002 (−0.03 to 0.03)	.90	0.05 (−0.03 to 0.12)	.22	−0.03 (−0.09 to 0.02)		.20

### Qualitative Results

#### Overview

**Figure 9:**
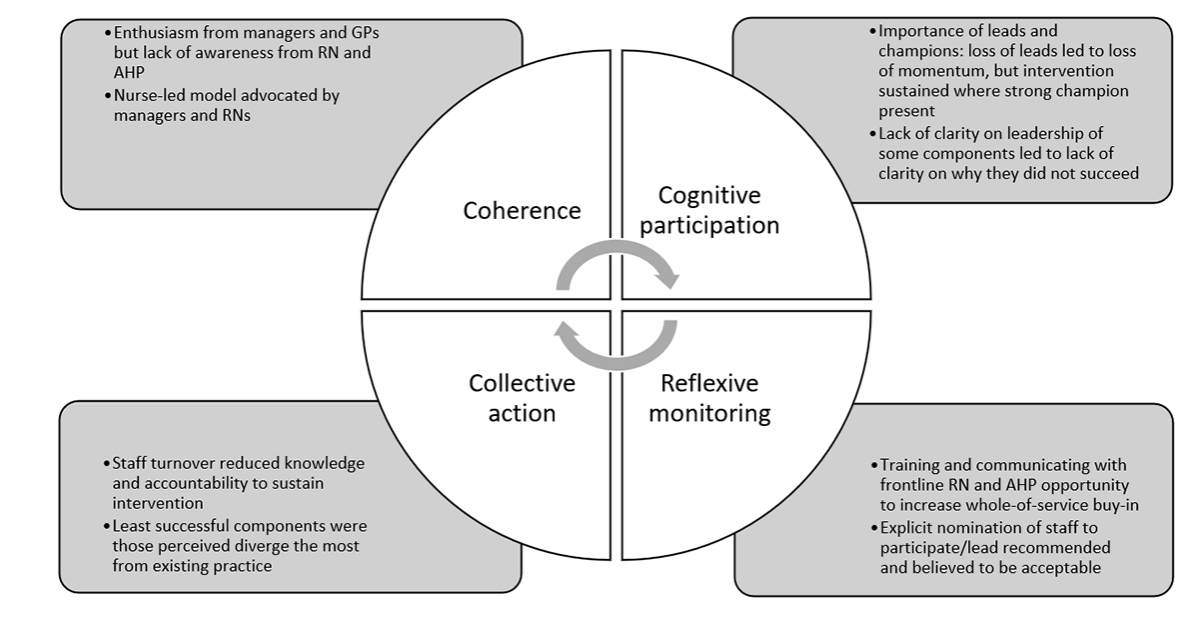
Thematic map of qualitative analysis informed by normalization process theory. AHP: Aboriginal health practitioner; GP: general practitioner; RN: registered nurse.

A total of 6 ACCHS staff members participated in an interview: managers (n=2, 33%), GPs (n=2, 33%), an RN (n=1, 17%), and an AHP (n=1, 17%), representing a little more than half of the staff members who had maintained continuous employment from before the implementation until after the end of the postintervention period. The qualitative interviews explored potential explanations for the quantitative findings through seeking to understand how ACCHS staff had experienced and perceived the implementation of the systems change and its impact both on the STI testing rates examined in the quantitative analysis and on everyday work practice. This allowed the research team to tease out which components of the systems change had or had not been successfully normalized, what factors had contributed to normalization, and how the successful or unsuccessful normalization of different components of the systems change explained key findings from the quantitative analysis. The coding framework consists of 4 NPT constructs: coherence (how participants make sense of the change), cognitive participation (engagement by participants), collective action (the participants’ work that has contributed to the intervention functioning), and reflexive monitoring (participant assessment of benefits and costs). Figure 9 captures the key insights from the qualitative data of relevance to each of the 4 NPT constructs.

#### Coherence

Managers and GPs displayed enthusiasm about the electronic prompts and automated pathology sets, but the RN and the AHP, whose roles did not require them to use the prompts and test sets, demonstrated low awareness. Communication to frontline staff about the systems change seemed to have been inadequate to instill a shared understanding of its importance and value. However, participants who demonstrated a high awareness of the systems change identified that the systematized incorporation of age-based STI testing into the health assessment had had a broader impact on organizational norms around sexual health in routine practice, with several participants identifying this as a potential explanation for the observed increase in STI testing:

Previously it was an individual clinician who may or may not have decided this patient should be tested. Like there was no organizational-wide policy or...expectation that clinicians would be doing that.... Our [health assessments] were changed to include the sexual health testing for people in the targeted age range. So that lifted sexual health screening from sort of some nebulous thing that people may or may not do...to something that was very much at the forefront and was discussed at clinical meetings. It really gained a life of its own which it had never had previously.

Participant 1, manager

Participants expressed appreciation for RNs and AHPs, but the RN and AHP participants felt that non-GP clinical roles were not leveraged to their full potential and that this had limited the successful implementation of the systems change. All participants recognized the importance of the “soft” skill sets of RNs and AHPs in patient engagement. Adding to this with enhanced clinical autonomy and a nurse-led model of care via credentialing RNs and AHPs to complete STI screens independently was broadly supported by staff at all levels. Participants considered the nurse credentialing component of the package and the presigned pathology forms component to be strongly interlinked. However, whereas participants reported strong support for the credentialing component, the pathology forms met resistance among the GP workforce, although the RN participant was supportive of the component:

One thing I encounter when I was working here is things like, just ordering pathology form, like STI check, for example, if they can allow the nurses to do that, you know. Because sometimes people just come in.... They are asymptomatic.... But they don’t want to see the doctor...if I’m given the autonomy to practice, I could just give the, print out a pathology form and order it.... If the nurses are allowed to do, to order things like that, STI check, then it may improve the STI screening.

Participant 6, RN

The underuse of the non-GP personnel was also apparent in participant discussions of data reporting. An increased transparency of data was perceived positively, and several participants described an increased awareness of trends in practice data as motivating for the workforce. However, GPs and managers again had much greater awareness of the changes that had been made than the RN and AHP participants, although the RN and AHP participants felt that strengthening communication with frontline staff would increase whole-of-service commitment to improving outcomes. Participants did not identify any linkages between the improvements made to data analysis capacity and the quantitative findings.

#### Cognitive Participation

All participants perceived leads and champions as vital to moving the systems change forward, which was most evident in relation to the electronic prompts and automated pathology sets. Strong leadership encouraged early adoption among the GP workforce, but the departure of people in key lead or champion roles led to this action being deprioritized among some newer GPs. It was sustained consistently among GPs who had been present before the development of the package, but only implemented ad hoc for newer GPs after the departure of a key GP champion partway through the study period. Participants perceived staff turnover as a barrier to normalizing this component and felt that the positive impact seen on STI testing rates within the health assessment was the result of the commitment of longer-term staff to this component of the systems change.

Participants were unclear who held responsibility for the credentialing of RNs and AHPs. Some RNs completed training, but this lack of clarity impeded the necessary systemic change to routine practice. The presigned pathology forms were successfully sustained in the prenatal setting owing to a strong GP champion, and there was interest from RNs service-wide, but outside of the prenatal setting, no individual GP had self-nominated as the signatory. The lack of GP commitment to this component also prevented the implementation of the credentialing component because RNs who had completed credentialing could not order tests without presigned forms. As the aim of these components was to enable RN- and AHP-led testing outside of GP consultations, this explains the lower increase in STI testing outside of the health assessment:

I think a GP champion and I’m gonna say the sexual health physician that should be full time in that sexual health area.... Apart from the fact we have an inexperienced, untrained person in the job, there’s just not enough time left over to give it, to actually do all that; you know, that working one-on-one with people and following up, and talking to the GPs, and...yeah. So that’s what I’d say. A GP champion and a full-time sexual health RN or health worker.

Participant 1, manager

However, participants did not always expect champions to be senior clinicians or managers. The standardization of the health assessment was perceived to provide systemic support for AHPs to champion the inclusion of routine STI testing to GPs who may otherwise use their discretion to not include it, which further supports the systems change as the driver of increased testing within the health assessment. Participants expressed that this normalization of practice had partially addressed the concern regarding some newer or locum GPs not having implemented the EMR modifications:

If we’ve got good-quality, well-trained health workers, I think that’s a really big plus and a really good way for it to go forward, ’cause it doesn’t depend on the GP who might be a locum for 2 weeks: it depends on that health worker going, “Part of our [health assessment] is that we always do this comprehensive STI screen.”

Participant 4, GP

Although participants supported improvements in data reporting in principle, they almost universally perceived it as something that they were recipients of, rather than participants in. The interviews depicted an understanding of data reporting as a contribution to the service made by an unidentified other that would then inform participants’ work, rather than a reflection of the outcomes of their work that could meaningfully contribute to quality improvement. This perhaps explains why participants did not perceive a strong relationship between this component and the quantitative findings.

#### Collective Action

Considerable staff turnover and limited follow-up from the project team owing to travel restrictions imposed at the onset of the COVID-19 pandemic reduced knowledge of the systems change among service staff. This contributed to a reduction in the shared accountability required to sustain the components of the systems change that had not already been successfully normalized. The electronic prompts also required moderate digital literacy on the part of individual GPs, and frequent staffing changes and low numbers of long-term ongoing GPs prevented technological upskilling of the GP workforce as a whole:

’Cause we have so many doctors coming and going, we do have a couple of local GPs as well. GPs that have been here for years. You know, they’re here for 4 weeks, they’re away 4 weeks, back for 4 weeks. And then obviously, you know, we have locums which come and go, you know. They’re here for 2 weeks. They’re gone. And then you get the next one. So, I think it’s just basically having so many staff coming and going, and having the availability I guess to, to train them or to go over all that sort of stuff.

Participant 5, AHP

Of the 4 components, the presigned pathology forms component was perceived to diverge the most from existing practice, requiring cultural as well as process change. This finding was of interest to us because it did not reflect the outcomes of the initial collaborative development of the systems change. During the planning process, this component had been perceived by service staff as an “easy win,” but retrospectively it was seen by participants as a significant and extremely challenging change.

The enhanced data reporting was initially implemented successfully and sustained during the study period but was not sustained during the COVID-19 pandemic. However, participants perceived benefits to reinstating this component in a post–COVID-19 context. Participants recognized the normalization of STI testing as an ongoing investment in community health and continued to view this as an important priority even in the context of a recent and ongoing crisis that disrupted previously normalized practice.

#### Reflexive Monitoring

The electronic prompts and automated pathology sets had been proposed by staff as a strategy with high potential to reduce friction and minimize provider-end barriers to increasing comprehensive STI testing. However, although participants felt that the component had realized some of its potential among both existing staff of the service and some newcomers, and the quantitative findings indicate that it had successfully increased STI testing within the health assessment, the capacity for universal normalization was limited by EMR functionality requiring separate configuration for each individual GP user. Participants identified an opportunity to reconfigure the component so that it is implemented from the level of either the software vendor or the pathology level rather than on the practice level and believed that this greater systematization would enable the replicability and sustainability of the observed increase in STI testing:

Putting the prompt there, it’s easier to one-click for the GPs, to select it, and we will get all the tests that we need, and make sure that it gets reported correctly from the path lab. So we’ll add the STI testing, you know, thinking we’ll just go for chlamydia, get the urine. But having it on the [autopopulated] panel it says “syphilis,” so, therefore, it goes back to either the pathology collector or the nurse, or health worker; they’ll immediately draw the blood...I think that’s worthwhile.

Participant 2, manager

The credentialing component was not integrated into regular practice, explaining our finding of limited increase in overall testing across all consultation types. Participants identified value in introducing a defined clinical educator role to support the training of RNs and the alignment of clinical care with policy. Managers reported that the lack of support from GPs for the presigned pathology forms component dampened the enthusiasm of RNs but believed that the explicit delegation of a GP by management to presign forms would be acceptable to the GP workforce.

Participants perceived a link between the data component and existing and prospective internal communications mechanisms, which they identified as an opportunity to strengthen and sustain the systems change. Recommendations included incorporating data reporting into existing meeting agendas and building on existing channels to streamline communication between management and frontline staff (eg, via a staff newsletter).

## Discussion

### Principal Findings

This study provides evidence for the first time that optimizing the health assessment is an effective strategy to increase STI testing in the ACCHS setting. It also contributes to the evidence base on the use of electronic prompts and automated pathology sets to increase STI testing, demonstrating that these are effective in the ACCHS setting and acceptable to ACCHS staff. Our qualitative data show that across all 4 components of the systems change, the electronic prompts and automated pathology sets had the highest level of integration and normalization into routine practice, resulting in a substantial and sustained increase in STI testing within the health assessment seen in our quantitative data.

In our evaluation, we observed an increase in overall STI testing integrated into the health assessment in the period when the electronic prompts and automated pathology sets were introduced, from 16% (38/237) in year 1 to 42.9% (94/219) in the intervention period. Although an increase in syphilis testing was observed both within and outside of the health assessment, this was not accompanied by an increase in CT/NG tests outside of the health assessment. As no effect was observed for the internal control, and the intervention coincided with an immediate increase in STI testing, we can conclude that the increases are likely to have been the result of the electronic prompts and automated pathology test sets implemented as part of the systems change. The increase in STI testing generally was primarily seen within health assessment consultations, and the electronic prompt was the only component of the systems change to focus directly on the health assessment, with other components focusing on other consultation types. The increase in syphilis testing outside of the health assessment occurred independently of an increase in tests for other STIs, indicating that this effect represents the inclusion of syphilis tests in existing STI test requests. This suggests that the electronic prompts and automated pathology test sets and their resulting normalization into routine practice were the components of the systems change responsible for the increase in STI testing observed within health assessment consultations and the increase in syphilis testing specifically.

The successful adoption of STI testing into the health assessment by providers and patients is likely to be due to the perceived acceptability and cultural safety of the strategy [57]. Age-based STI screening in routine medical consultations is perceived as acceptable by both GPs and their male and female patients [58-60], which may have contributed to strong support for this strategy among our interview participants. However, the electronic component of the systems change could have achieved greater reach if more health assessments had been conducted among young people. We found that only a quarter of the young people received a health assessment in both the pre- and postintervention periods, consistent with another study in the same context [17]. This meant that despite the increases in STI testing within the health assessment, we only saw a modest increase in STI testing overall, consistent with a previous CQI program involving enhanced data reporting and incentives that increased STI testing among young Aboriginal people within health assessments but not in routine medical consultations outside of the health assessment [61]. Fully realizing the potential of the electronic optimization of the health assessment to increase STI testing will require investment in strategies to increase the uptake of the health assessment itself. This will require investment in both provider-end systems change and better resourcing of health promotion to increase patient demand [62]. Currently, the health service participates in a health promotion program called Deadly Choices that provides nonfinancial incentives to patients for participating in a health assessment. The role of nonfinancial incentives in increasing health assessment uptake is the focus of a future paper.

Our interview participants valued the work done by champions to push the systems change forward and consistently advocated further investment in dedicated champions to advance project goals. However, the qualitative data also provided evidence of the vulnerabilities created when systems change relies on individuals rather than a sustained, collective, whole-of-service approach. Two components that aimed to increase STI testing outside of the health assessment—the credentialing of RNs and AHPs as well as the presigning of pathology request forms—were not successfully implemented, which explains why STI testing increased substantially within the health assessment but only modestly outside of it. In the primary care setting, RNs tend not to hold positions of equal power to GPs or engage in shared decision-making, and in the Australian context specifically, the inability of RNs to claim reimbursement from Medicare for clinical services has been identified as a significant barrier to increasing shared care [63]. Previous research examining RN-led cervical screening using a model similar to the one discussed in this paper identified medicolegal implications in GPs using their provider number to authorize tests ordered by RNs because there is no way to identify the clinician responsible for requesting an individual test [64]. Our interview participants attributed the unsuccessful normalization of these 2 components primarily to the loss of momentum caused by the departure of key champions. The value of champions in driving forward innovations has been recognized in ACCHS and other primary care settings [65,66], and Aboriginal leadership, in particular, remains an often untapped resource [67]. However, the *National Framework for Continuous Quality Improvement in Primary Health Care for Aboriginal and Torres Strait Islander People 2018-2023* [68] advocates for systems change to be championed at the organizational level rather than by individual staff and recommends that leadership encourage a culture of “CQI is everyone’s business.”

We also found that a higher proportion of health assessments conducted with men included an STI test in both the pre- and postintervention periods, compared with women, and the increase in the proportion of health assessments that included an STI test in the postintervention period was greater for men than for women. In addition, the proportion of health assessments inclusive of a chlamydia test that had a concurrent syphilis test increased more substantially in the postintervention period for men than for women. Although other studies have instead found that the uptake of STI testing overall is higher in young Aboriginal women [1,17,69-73], our previous work has identified gender parity in STI testing in the context of the health assessment [74]. One potential explanation for this identified in the qualitative data was the impact of standardization resulting in routine tests being offered during the health assessment that clinicians may have previously used their discretion to skip. Qualitative research with Aboriginal men has identified not only a reluctance to proactively access health care [75] but also strongly held values for being a responsible sexual partner [76], highlighting the importance of health assessment in providing routine, comprehensive STI testing to young Aboriginal men who may not otherwise seek out testing.

Normalizing STI testing into routine practice is the cumulative product of multiple small and interrelated changes [77], and our qualitative findings support the value of this model for promoting changes in clinical practice related to STI testing owing to the varied and individual needs of clinicians targeted by systems change [78]. Collaboratively designing this systems change ensured high acceptability even of those components that were not successfully implemented or sustained. Previous literature has found that RN-led testing increases STI testing in primary care, while also noting difficulty in sustaining the required changes to the supporting infrastructure and processes [77]. Although some RNs perceive GPs’ reluctance to share care to be a barrier to implementing RN-led testing [39], related research has found that GPs support RN-led testing and believe that it is suitable for their skill set [79]. Our findings suggest potential for more successful future implementation of these components of the systems change, with appropriate investment in ongoing engagement of staff at all levels. Future work using NPT may benefit from examining the elements of collaboration associated with successful systems change and identifying what may satisfy expectations to achieve normalization.

Our evaluation has a few limitations to consider when interpreting the findings. First, the qualitative data were collected over a longer period than was planned after the conclusion of the postintervention period owing to the impacts of the COVID-19 pandemic on the participating ACCHS. Although it is also a standard expectation that qualitative findings be recognized as nongeneralizable owing to the small samples typical in qualitative research, the qualitative sample size represented more than half of the staff who had continued employment throughout the study period, which supports the value of incorporating these findings into the analysis as a whole. Second, our quantitative analysis used a before-and-after design to evaluate the effectiveness of the intervention, which may be subject to temporal biases but was strengthened using an ITS analysis with a 2-year before period (12 time points) and an internal control. Third, we were unable to evaluate which GPs installed the prompts and automated pathology sets or how frequently they were activated. Finally, the strategy was evaluated at 1 health service; therefore, the findings may not be generalizable to all NSW ACCHSs. The use of the NPT framework is a strength of this study because a systematic review has identified that NPT strengthens analysis of implementation processes [28].

### Conclusions

We have shown that the introduction to the EMR of an electronic prompt to encourage GPs to offer age-based STI testing and a shortcut to autopopulate a pathology request for a comprehensive STI test is an effective and acceptable strategy to increase both integration of STI tests into the health assessment and the inclusion of syphilis testing in STI tests completed within the health assessment in the ACCHS setting. Future systems change should also incorporate strategies to increase the uptake of the health assessment among the target population and work to better promote whole-of-service engagement and accountability to increase normalization. Future work exploring the suitability of related interventions for other sites should continue collaboration with ACCHS staff to develop locally customized solutions.

## Abbreviations

ACCHS: Aboriginal Community Controlled Health Service
AHP: Aboriginal health practitioner
CQI: continuous quality improvement
CT: *Chlamydia trachomatis*
EMR: electronic medical record
GP: general practitioner
GRHANITE: Generic Health Network Information Technology for the Enterprise
ITS: interrupted time series
NG: *Neisseria gonorrhea*
NPT: normalization process theory
NSW: New South Wales
RN: registered nurse
STI: sexually transmitted infection
